# Indistinguishable odour enantiomers: Differences between peripheral and central-nervous electrophysiological responses

**DOI:** 10.1038/s41598-017-09594-3

**Published:** 2017-08-21

**Authors:** Sophia C. Poletti, Annachiara Cavazzana, Cagdas Guducu, Maria Larsson, Thomas Hummel

**Affiliations:** 1 Smell & Taste Clinic, Department of Otorhinolaryngology, ‘Technische Universität’, Dresden, Germany; 2 Stockholm University, Department of Psychology, 106 91 Stockholm, Sweden; 3 Dokuz Eylul University, Department of Biophysics, 35340 Balcova, Izmir Turkey

## Abstract

The ability of humans to discriminate enantiomeric odour pairs is substance –specific. Current literature suggests that psychophysical discrimination of odour enantiomers mainly depends on the peripheral processing at the level of the olfactory sensory neurons (OSN). To study the influence of central processing in discrimination, we investigated differences in the electrophysiological responses to psychophysically indistinguishable (+)- and (−)- rose oxide enantiomers at peripheral and central-nervous levels in humans. We recorded the electro-olfactogram (EOG) from the olfactory epithelium and the EEG-derived olfactory event-related potentials (OERP). Results from a psychophysical three alternative forced choice test indicated indistinguishability of the two odour enantiomers. In a total of 19 young participants EOG could be recorded in 74 and OERP in 95% of subjects. Significantly different EOG amplitudes and latencies were recorded in response to the 2 stimuli. However, no such differences in amplitude or latency emerged for the OERP. In conclusion, although the pair of enantiomer could be discriminated at a peripheral level this did not lead to a central-nervous/cognitive differentiation of the two stimuli.

## Introduction

The ability to discriminate molecular structures from its mirror image, the so-called chiral recognition of substances, belongs to the basic principles of biological activity^1^. Except for the optical activity, odour enantiomers hold identical physical and chemical features. Besides differences in olfactory perception, diverse effects of enantiomers have also been well described in the context of taste perception^2^ and drug response^3, 4^. In the 90 s olfactory receptors have been identified as proteins, i.e. chiral molecules^5^, for which reason the interaction between enantiomers and receptors was assumed to be enantio-selective. Therefore, any differences in odour perception were understood to result from the selectivity of olfactory receptors at the level of the olfactory epithelium^6, 7^. However, not only distinguishable but also identically smelling or indistinguishable enantiomeric odour pairs have been described in humans and non-human primates^8^ highlighting an unpredictable relationship between odour structure and odour perception.

The olfactory bulb represents a central neural source which seems to be critical for odour discrimination. Information from the olfactory sensory neurons (OSN) is represented in the activity of olfactory bulb glomeruli^9–12^. Linster *et al*. demonstrated in rats the predictability of odour enantiomer discrimination from their evoked patterns of neural activation in the olfactory bulb^13^. A rather biological approach explaining the discriminability of only some odour enantiomers might be the occurrence of both optical isomers in nature. In analogy to the immune system there is increasing evidence for the mammalian OSNs to increase its receptor expression following repeated exposure to olfactory stimuli^14–16^. Thus, it has been hypothesized, when both optical isomers occur in a natural environment, the appropriate enantio-selective receptors will enable the differentiation of the pair of odours.

Given the lack of clarity in the neurophysiological mechanism of odour discrimination, we set out to explore the differences in peripheral and central representations of indistinguishable rose oxide enantiomers by recording electro-olfactograms (EOG) from the olfactory epithelium and central-nervous, EEG-derived olfactory-event-related potentials (OERP) from the scalp. We hypothesized that indistinguishable odour enantiomers show electrophysiological differences at the level of the OSNs (demonstrating enantio-selectivity regardless of the psychophysical discriminability) while the central neural representation exhibits similar response patterns which would be the basis for similar behavioural responses or psychophysical indistinguishability.

## Results

### Behavioural data

**Figure 1:**
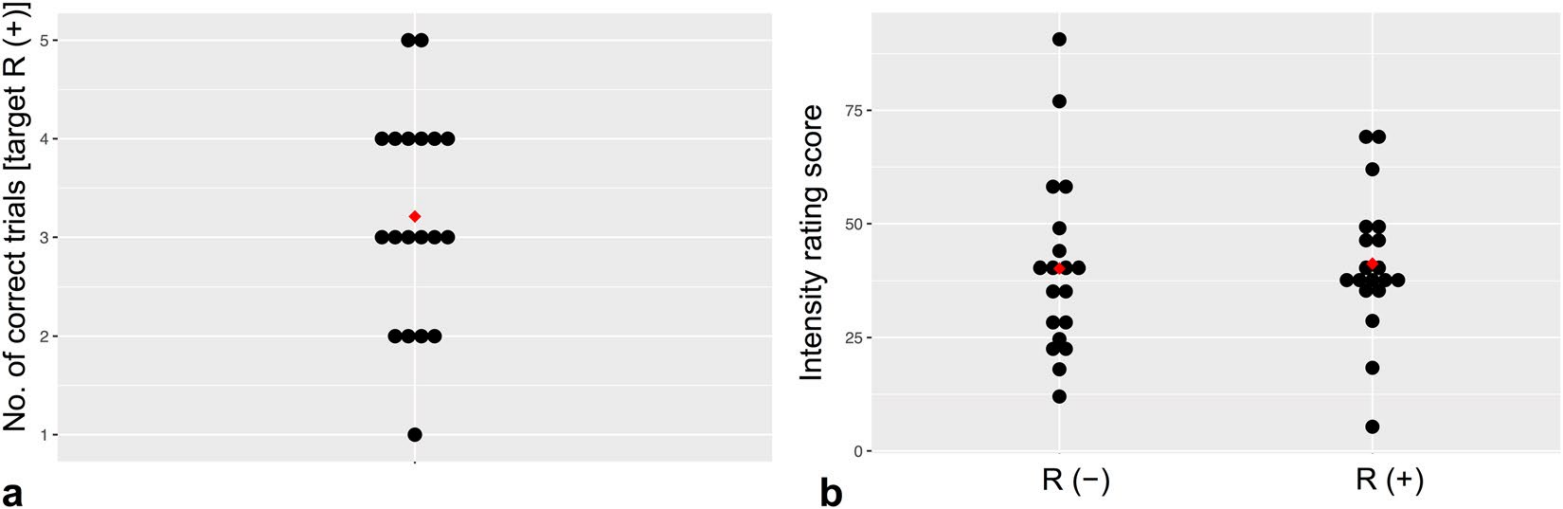
Results from behavioural test. (**a**) 3-AFC discrimination test. The black dots show each participant with their number of correct trials out of 9; the red dot represents the mean of correct trials. (**b**) Intensity ratings for (+)-rose oxide and (−)-rose oxide. The black dots show the intensity rating of each participant. The red dot indicates the mean of the intensity ratings of (+)-rose oxide and (−)-rose oxide.

All subjects showed a normal sense of smell with an average TDI score of 38 points (SD: 3.3, ∅ scores: threshold (T): 10 (SD: 2), discrimination (D): 13 (SD: 2), identification (I): 15 (SD: 1)). The average discrimination performance for the target odour was 3.2 (SD: 1.01). Applying a one sample t-test, this result did not differ significantly from chance level (*t* [18]) = 0.85, *p* = 0.41). Regarding the intensity rating of R (+) and R (−) no significant differences emerged between the two enantiomers (∅ intensity R (+): 41 (SD: 15.4), R (−): 40 (SD: 19.9); *t* [18]) = −0.35, *p* = 0.73) as showed by the paired sample t-test. These results indicated psychophysical indistinguishability of R (+) and R (−) (Fig. 1a and b).

### Electrophysiological data

#### EOG

**Figure 2:**
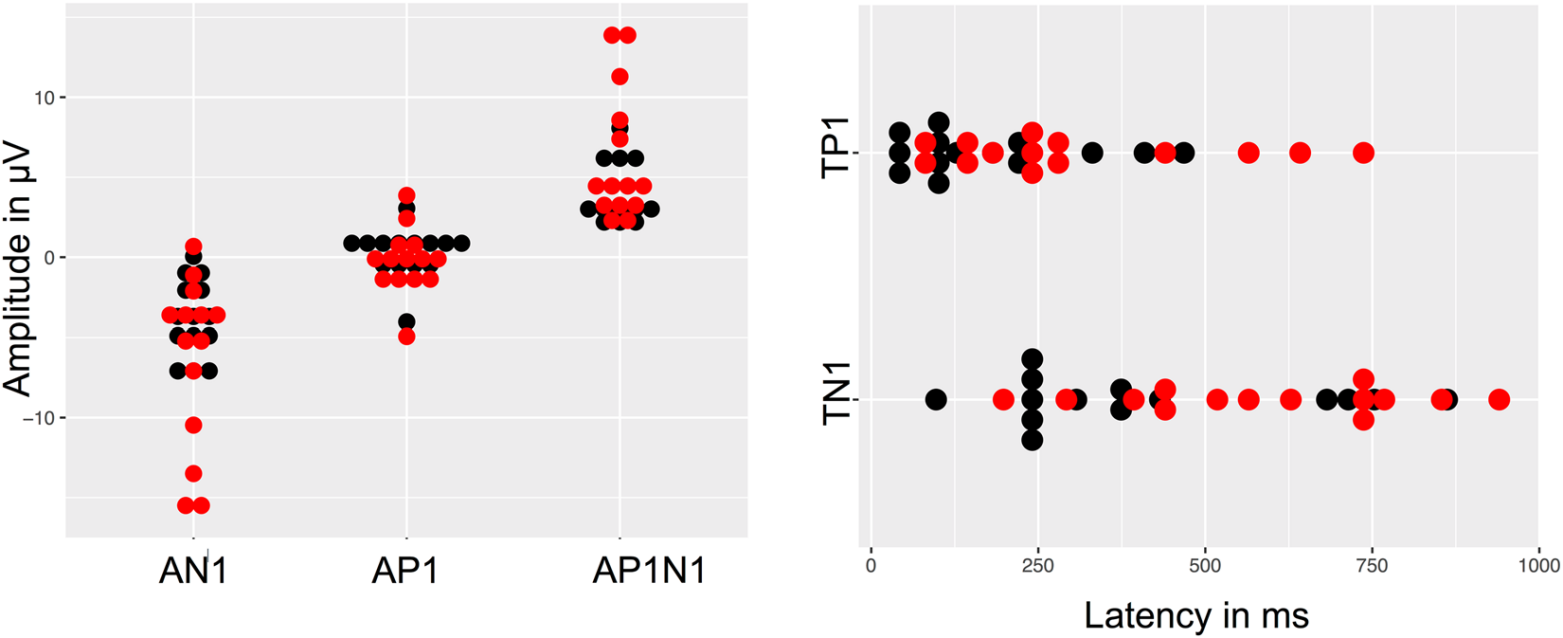
Electrophysiological responses at the olfactory epithelium in response to the odour enantiomers of (−)- rose oxide and (+)- rose oxide. The black and the red dots show responses to R (−) and R (+) respectively. (**A**) Mean EOG amplitudes (base-to-peak) AN1 and (peak-to-peak) AP1N1 of (−)- and (+)-rose oxide differ significantly (*p < 0.05), with higher amplitudes in response to (+)-rose oxide. (**B**) EOG peak latencies TP1 and TN1 of each participant in response to (−)- and (+)-rose oxide. Significantly higher mean latencies TP1 and TN1 were obtained after (+)-rose oxide odour stimulation (*p < 0.05).

**Figure 3:**
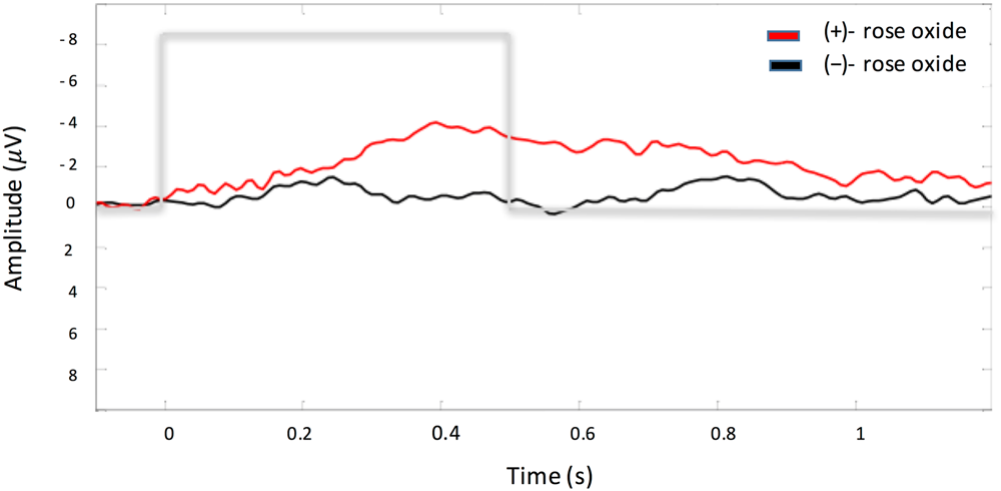
Grand average of EOG in response to (+)-rose oxide (red line) and (−)-rose oxide (black line). Grey line presents stimulus starting from 0 s with a sharp onset and duration of 0.5 s.

EOG recordings could be obtained in 14 out of 19 (74%) participants. When comparing the electrophysiological responses to R (+) and R (−) – by using a paired sample t- test - there were significant differences in EOG amplitudes AN1 (*t* [13] = −2.29, *p* = 0.04) and AP1N1 (*t* [13] = 2.39, *p* = 0.03) with higher amplitudes obtained following R (+) stimulation (base-to-peak amplitude AN1 (in μV): R (+) 6.42 (SD: 5.3), R (−) 3.77(SD: 2.4)); peak-to-peak amplitude AP1N1 (in μV): R (+) 6.21 (SD: 4.1), R (−) 4.07 (SD: 1.9). Further, significant differences in the peak latencies TP1 (*t* [13] = 2.56, *p* = 0.024) and TN1(*t* [13] = 3.39, *p* = 0.005) were recorded with longer latencies found in response to R (+) (latencies: TP1 (in ms): R (+) 307 (SD: 208), R (−) 179 (SD: 137); TN1 (in ms): R (+) 589 (SD: 219), R (−) 415 (SD: 239)) (Figs 2A and B and 3).

#### OERP

**Figure 4:**
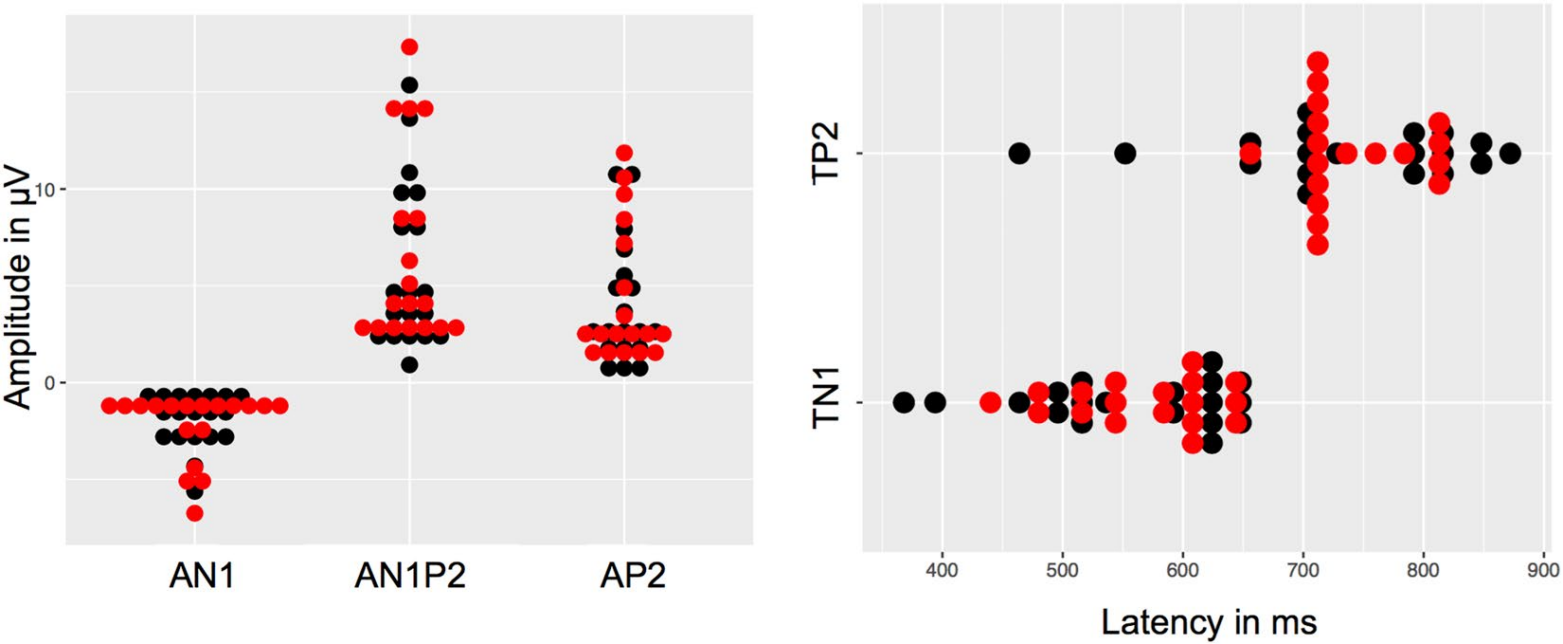
OERP representing cortical responses to (+)-rose oxide and (−)-rose oxide. The black and the red dots represent each participant’s electrophysiological response to R (−) and R (+). (**A**) Mean values (in μV) of AN1, AN1P2 and AP2 amplitudes showing no significant differences between (+)-rose oxide and (−)-rose oxide. (**B**) Mean latencies (in ms) TN1 and TP2 in response to (+)-rose oxide and (−)-rose oxide with no significant differences.

**Figure 5:**
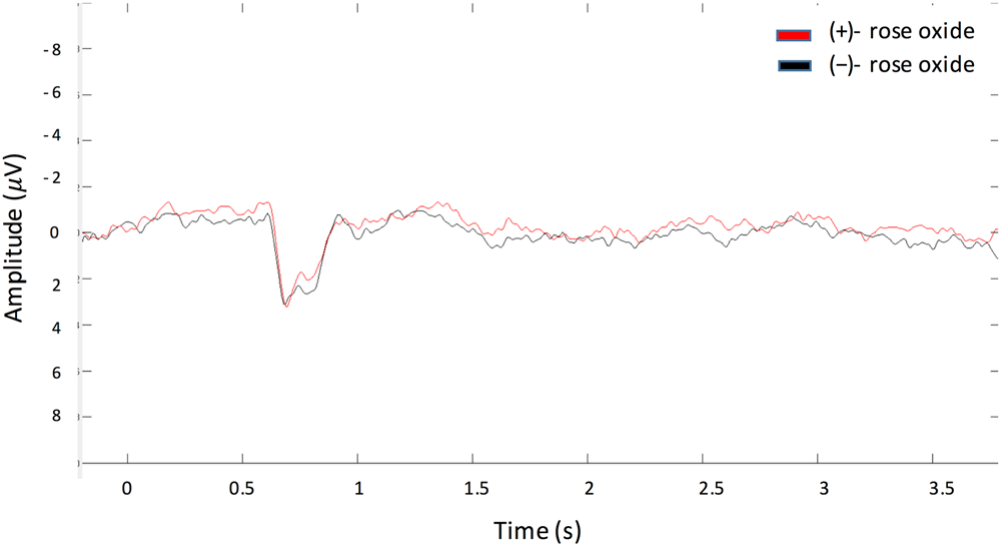
Grand average of OERP in response to (+)-rose oxide (red line) and (−)-rose oxide (black line).

OERPs in response to the enantiomers of rose oxide could be recorded in 18 out of 19 (95%) participants. Regarding amplitudes (AN1 (in μV): R (+) 2.29 (SD: 1.8), R (−) 1.94 (SD: 1.4), AP2: R (+) 4.42 (SD: 3.5), R (−) 4.08 (SD: 3.2), AN1P2: R (+) 6.71 (SD: 4.9), R (−) 6.01 (SD: 4.3)) and latencies (TN1 (in ms): R (+) 567 (SD: 62), R (−) 551 (SD: 87), TP2: R (+) 739 (SD: 47), R (−) 728 (SD: 103)) no significant differences emerged between the two enantiomers by means of paired sample t-test (all *p*_*s*_ > 0.05) (Figs 4A and B and 5).

## Discussion

The results of this study demonstrate that (1) psychophysically indistinguishable odour enantiomers show differences in peripheral response (EOG) but (2) no differences in central-nervous responses (OERP). These differences in EOG at the level of the OSN reveal distinct neuronal activation pattern of OSNs representing enantio-selectivity of the receptors for psychophysically indistinguishable odour enantiomers. Contrary to the assumptions of Laska and colleague, enantio-selectivity of olfactory receptors is given in same smelling odour enantiomers and therefore enantio-selectivity of OSNs seems not to predict the ability of humans to psychophysically discriminate molecular structures from its mirror image^7, 8, 17^.

Studies on the mechanism of olfactory perception and discrimination mainly focus on the peripheral interaction between molecules and receptors assuming to be the main locus for discrimination. For example, Laska *et al*. proposed that the combined presence of isopropenyl and methyl groups in specific positions of odour enantiomers allows their psychophysical discrimination^18^. Poivet *et al*. chose a different approach emphasizing biological function over chemical structures. They demonstrated the importance of topographical polar surface area of molecules in odour discrimination^19^.

Without a systematic understanding of odour detection and discrimination at the periphery it is difficult to imagine how central brain areas process the sensory input to generate perception. OSNs express one type of odorant receptors^20–22^ with those homologous for one receptor type converging onto a relatively small number of glomeruli within the OB^23–25^. The role of the OB as part of the central-nervous olfactory brain structures in odour discrimination was highlighted by Linster *et al*. in rats^13^ showing similar neural OB representation patterns for indistinguishable and distinct glomerular activation areas for distinguishable enantiomeric odour pairs. However, McBride *et al*. questioned these findings by demonstrating that surgical lesion of glomerular regions activated by odour enantiomers does not seem to influence discriminatory ability^26^. Because olfactory sensory information appears to be represented one-to-one in the activity of olfactory bulb glomeruli and we believe that - regardless of the psychophysical distinguishability - odour enantiomers show enantio-selectivity at the OSN level, we expect even higher central olfactory regions than the OB to be responsible for odour enantiomer discrimination. As shown in our study, psychophysically indistinguishable odour enantiomers of rose oxide showed differences in neural response at the peripheral level whereas no differences emerged in the activation pattern at the cortical level. We considered that differences in concentration of R (+) and R (−) (0.0275 vs 0.02) - at which odour enantiomer indistinguishability and lack of trigeminal stimulation was demonstrated - could have contributed to differences in EOG potentials^27^. However, in this case, we would have expected similar OERP differences at the central level in response to the same odour enantiomer concentrations which did not appear^28^. Hence, our findings indicate that indistinguishable odour enantiomers evoking similar cortical electrophysiological patterns in humans may result in similar behavioural responses. Consequently, a structure-based encoding seems to be present in the periphery (OSN) but the quality-based encoding shows no direct relation to structural configuration. Although we can draw no conclusion as to where the distinct information from the OSNs to R (+)- and R (−) converged or even dissolved at the central level leading to similar OERP, the following structures may be relevant to that process. The primary olfactory cortex (especially the piriform cortex) receives all direct bulbar input^29, 30^ and also sends feedback projections to the OB^31^ providing many ways for the modulation of sensory processing. Higher projections from these primary regions then converge on ‘olfactory regions’ of the orbitofrontal cortex where a ‘one-to-one’ input is lacking. Therefore, these highly processed representations seem to transcend the original sensory input and represent not only structural based codes but also include information, e.g., on previous experience.

Coding mechanisms of odour quality and odour structure in the human piriform cortex have been thoroughly investigated by Gottfried and colleagues^32^. While the anterior piriform cortex encodes structures of odours, the posterior region is more related to encoding of odour quality. The structure based coding appears to represent the sensory information from the OB. But a structure independent coding based on the quality of an odour, which depends on learning and experience, could explain why some odour enantiomers are perceived differently and some not. In return, the piriform cortex could therefore represent the region where distinct peripheral electrophysiological responses to R (+)- and R (−) are finally interpreted resulting in similar OERP reflecting psychophysical indistinguishability. In order to further corroborate our data, we decided to explore whether a couple of easily distinguishable odor enantiomers could evoke different OERP patterns. Limonene^7^ [(+)- limonene and (−)- limonene] was tested in a group of 12 healthy participants by applying the same experimental procedure (see the Supplementary Information for an overview of the methodology and the results). In line with literature, participants were able to psychophysically discriminate between the two enantiomers of limonene^7^ and this result was also reflected in the brain by showing significant distinct OERP patterns in response to (+)- limonene and (−)- limonene. With this finding, we underline our hypothesis on the importance of the central rather than the peripheral processing of psychophysical discriminability.

In conclusion, we demonstrated that odour enantiomers evoking different responses at the level of the olfactory epithelium but similar cortical response patterns resulted in similar behavioural responses. Hence, a structure-based encoding seems to be present in the periphery which is not necessarily translated into a central nervous electrophysiological or behavioural response.

## Material and Methods

### Participants

A prospective study was conducted at the Smell and Taste Clinic at the Department of Otolaryngology of the “Technische Universität” (TU) Dresden. The study was performed according to the Declaration of Helsinki and approved by the Ethics Committee of the Medical Faculty at the TU Dresden (application number: EK361082016). All experiments were undertaken after subjects had provided written informed consent. Only participants between the age of 18 and 40 years with a normal sense of smell ascertained by means of Sniffin’ Sticks^33^ were included in the study. Further, nasal endoscopy was performed in all participants to evaluate the endonasal anatomy in the area of the olfactory cleft. We finally included only those subjects with a clearly visible olfactory cleft in order to enable easy placement of the EOG electrode without causing much discomfort to the participants. The following exclusion criteria applied: pregnancy, previous head trauma leading to unconsciousness, chronic/acute rhinosinusitis, neurological diseases, any systemic disease associated with smell disorders like chronic renal failure or thyroid disorders, and impaired sense of smell. Participants were instructed to only drink water one hour prior to the experiment and not to wear any scented products on the day of testing. Finally, a total number of 19 out of 26 tested participants (14 women, 5 men) with an average age of 25 years (range: 20 to 36 years, SD: 3.5) were included in the study.

### Enantiomers and trigeminality

A pair of indistinguishable odour enantiomers was used as stimulus^7, 34^: (+)-rose oxide (Fisher Scientific, CAS 16409-43-1) and (−)-rose oxide (Sigma-Aldrich, CAS, 16409-43-1). Starting from concentrations used in the study of Li *et al*.^34^ at which enantiomers were indistinguishable (11.1% (+)-rose oxide and 8.3% (−)-rose oxide), we further diluted the odours using 1,2- propandiol (Sigma-Aldrich, CAS 57-55-6). First, a lateralization task was employed^35, 36^. This was based on the repetitive presentation (n = 20) of odours to the left or right nostril the result of which depends on trigeminal sensitivity. This pilot study was performed in an independent sample of 10 participants in order to determine the trigeminal activity of the two odorants. If participants are not able to correctly localize the stimulated nostril we considered the odorant to not elicit any major trigeminal activation. The information was needed to (i) avoid behavioural discrimination based on the trigeminal component of the odour and (ii) to clearly obtain responses in response to olfactory but not to trigeminal stimulation when recording EOG.

The final concentrations used for the study were 0.0275% (+)-rose oxide (R (+)) and 0.02% (−)-rose oxide (R (−)). Based on an independent pilot study in 15 subjects, a three-alternative force choice (3-AFC) demonstrated that at these concentrations the odours were clearly perceivable, indistinguishable and rated as iso-intense (see the “behavioural testing” section for details on the 3-AFC). Odours were always presented to one nostril using a computer-controlled air-dilution olfactometer (OM6b; Burghart, Wedel, Germany) capable of delivering odorants without altering mechanical or thermal conditions inside the nasal cavity.

### Behavioural testing

The ability to discriminate between the two enantiomers was assessed using 9 trials of a 3-AFC discrimination test. The participants had to discriminate the target odour (in our case R (+)) from two identical non-target odours (R (−)). The stimulus duration was set at 500 ms with an inter-stimulus interval (ISI) of 6 s and approximately 20 s between presentations of each triplet. Subsequently, participants were also asked to provide ratings of odour intensity using a computerized visual analogue scale (VAS) ranging from 0 to 100 units (0 - “stimulus not perceived” to 100 - “very intense”). If no significant difference in intensity ratings emerged and the results from the 3-AFC test (9 trials) did not differ from chance level, we considered R (−) and R (+) to be psychophysically indistinguishable.

### Electrophysiological data acquisition

#### Olfactometer settings

Before exposing participants to the odorants, a specific breathing technique (velopharyngeal closure)^37^ was trained in order to avoid respiratory flow inside the nasal cavity. The site of chemical stimulation and EOG recording were chosen according to the endonasal anatomical proportions as described above. For odorous stimulation an olfactometer (OM6b; Burghart, Wedel, Germany) was employed^37, 38^. Stimuli were embedded in a constantly flowing air stream of controlled temperature (36.5 °C) and humidity (80% relative humidity) which was directed into the nasal cavity by means of a Teflon® tubing (8 cm length, 4/2 mm outer/inner diameter). Total flow rate was set at 6 L/min. With a sharp stimulus onset required, two thirds of the maximum stimulus concentration were reached at the olfactometer’s outlet within 20 ms^37^. During experiments subjects were seated comfortably in an air-conditioned room. White noise of approximately 50 dB SPL was used to mask clicking sounds associated with odour presentation. Further, participants were instructed to perform a tracking task on a video monitor during stimulation to keep them in an awake and vigilant state during the recordings^39^.

#### EOG, EEG settings

EOG was recorded by means of a tubular electrode (outer diameter 0.8 mm) filled with Ringer-agar (1%) which contained a silver-chlorided silver wire^37, 40^. Each electrode was freshly prepared less than 3 h before the experiment. For reference, an electrode normally used for EEG recordings (sintered Ag/AgCl electrode) was placed on each earlobe, two ground electrodes on each mastoid and a fifth electrode was placed above the right eyebrow to track vertical eye-blinks. The EOG electrode was guided medial to the middle turbinate at the level of its insertion to the back of the nose until contact had been established between tip of the electrode and the mucosa. Hence, the tip of the electrode was located at the transition area of the posterior end of the olfactory cleft and the anterior wall of the sphenoid sinus where olfactory epithelium can be expected with a high probability^41^. The location of the electrode was again verified endoscopically (Richard Wolf, Knittlingen, Germany; 30° visual angle; outer diameter 1.9 mm) and final adjustments were undertaken under endoscopic control. The electrode was then stabilized by means of an adjustable clip on a frame similar to lensless glasses^42^.

Besides the odour enantiomers, CO_2_ (60% v/v; air liquid, Düsseldorf, Germany) was used as stimulant at concentrations which clearly produced trigeminally mediated sensations such as stinging, burning or tickling. Thirty presentations of each odour enantiomer, 15 CO_2_ stimuli and 15 blank stimuli with a duration of 500ms were randomly presented to each participant. The inter stimulus interval was set at 20 (±4) s during which odourless air was delivered. Participants were instructed to control their eye blinking as much as possible. After an EOG response had been established in response to stimulation with either R (−), R (+) or CO_2_, the recording session started. Potentials were amplified, filtered (band pass 0.01–30 Hz), and digitized (sampling frequency 125 Hz, recording segments of 8192 ms). For recordings, pre-analysis was applied using Letswave 5 software (http://www.nocions.org/letswave5/). To establish baseline, recordings started 200 ms prior to stimulus onset. Simultaneously to EOG recordings, OERP recordings were obtained from position Cz (referenced against linked earlobes) with a Ag Ag/Cl electrode (Grass E5SH, Astro Med, West Warwick, RI, USA) according to the international 10–20 system using an 8-channel amplifier (Nihon Kohden, Tokyo, Japan). The epochs were inspected by eye for any kind of artefacts (blinks, muscle contractions etc.). Epochs containing amplitudes of 100 µV or higher were discarded. After the artefact rejection process for the EOG recordings had been completed, 3 to 5 “clean” epochs were baseline corrected, filtered with a low-pass filter (15 Hz) and averaged in the time domain. After averaging, maximum EOG peak amplitudes (P1, N1, p1n1) and peak latencies (lp1, ln1) were measured for each individual. For the EEG, clean epochs were baseline corrected, also filtered with a low-pass filter (30 Hz) and averaged in the time domain. Then, the peak amplitudes (N1, P2, n1p2) and peak latencies (ln1, lp2) of the ERP obtained from the Cz electrode were measured using Letswave 5 software.

### Statistical Analysis

Using SPSS 23.0 (SPSS Inc. Chicago, Ill., USA) data were statistically analysed by means of t-tests for paired samples to investigate differences in electrophysiological responses between the two enantiomers. To evaluate whether odour enantiomer discrimination ability of the participants differed from chance level, t-tests were performed with the chancel level set at 3. The level of significance was defined as *p* < 0.05.

### Data Availability

The datasets generated during and/or analysed during the current study are available from the corresponding author on reasonable request.

## Electronic supplementary material

Indistinguishable odour enantiomers: Differences between peripheral and central-nervous electrophysiological responses
