# Supplementary material for: Indistinguishable odour enantiomers: Differences between peripheral and central-nervous electrophysiological responses
Source: Sci Rep. 2017 Aug 21;7:8978. doi: 10.1038/s41598-017-09594-3 (PMC5566401; doi:10.1038/s41598-017-09594-3)
Supplement: Supplementary file 1 — Indistinguishable odour enantiomers: Differences between peripheral and central-nervous electrophysiological responses [file 41598_2017_9594_MOESM1_ESM.pdf]

Indistinguishable odour enantiomers: Differences between peripheral and central-nervous electrophysiological responses

Sophia C. Poletti[1,+], Annachiara Cavazzana[1,2,+], Cagdas Guducu[1,3], Maria Larsson[2], Thomas Hummel[1,*]

[+]these authors share first authorship for contributing equally

[1]Smell & Taste Clinic, Department of Otorhinolaryngology, 'Technische Universität', Dresden, Germany

[2]Stockholm University, Department of Psychology, 106 91 Stockholm, Sweden,

[3]Dokuz Eylul University, Department of Biophysics, 35340 Balcova, Izmir, Turkey

*Correspondence to: Thomas Hummel, MD, Smell & Taste Clinic, Department of Otorhinolaryngology, TU Dresden, Fetscherstrasse 74, 01307 Dresden, Germany; Ph +49-351-458-4189; F +49-351-458-4326; E-mail: thummel@mail.zih.tu-dresden.de

Discriminable odor enantiomers

Material and Methods

Participants

A prospective study was conducted at the Smell and Taste Clinic at the Department of Otolaryngology of the "Technische Universität" (TU) Dresden. The study was performed according to the Declaration of Helsinki and approved by the Ethics Committee of the Medical Faculty at the TU Dresden (application number: EK361082016). All experiments were undertaken after subjects had provided written informed consent. Only participants between the age of 18 and 40 years with a normal sense of smell ascertained by means of Sniffin' Sticks[1] were included in the study. The following exclusion criteria applied: pregnancy, previous head trauma leading to unconsciousness, chronic/acute rhinosinusitis, neurological diseases, any systemic disease associated with smell disorders like chronic renal failure or thyroid disorders, and impaired sense of smell. In addition, only participants who were clearly able to discriminate the two enantiomers were included in the study (i.e., with a discrimination score > 4 out of 9). Participants were instructed to only drink water one hour prior to the experiment and not to wear any scented products on the day of testing. A total number of 12 participants (6 women, 6 men) with an average age of 27.2 years (range: 21 to 40 years, SD: 5.1) was tested.

Odor stimuli and delivery

A pair of distinguishable odour enantiomers was used as stimulus[2]: (+)-limonene (50% v/v) (Sigma-Aldrich, CAS 5989-27-5) and (−)-limonene (50% v/v) (Sigma-Aldrich, CAS, 5989-54-8). Odours were always presented to one nostril using a computer-controlled air-dilution olfactometer (OM6b; Burghart, Wedel, Germany) capable of delivering odorants without altering mechanical or thermal conditions inside the nasal cavity. Stimuli were embedded in a constantly flowing air stream of

controlled temperature (36.5° C) and humidity (80% relative humidity) which was directed into the nasal cavity by means of a Teflon® tubing (8 cm length, 4/2 mm outer/inner diameter). Total flow rate was set at 6 L/min. With a sharp stimulus onset required, two thirds of the maximum stimulus concentration were reached at the olfactometer's outlet within 20 ms. During experiments subjects were seated comfortably in an air-conditioned room. White noise of approximately 50 dB SPL was used to mask clicking sounds associated with odour presentation. Further, participants were instructed to perform a tracking task on a video monitor during stimulation to keep them in an awake and vigilant state during the recordings. Before exposing participants to the odorants, a specific breathing technique (velopharyngeal closure) was trained in order to avoid respiratory flow inside the nasal cavity. Thirty presentations of each odor enantiomer with a duration of 500ms were randomly presented to each participant. The inter stimulus interval was set at 20 (±4) s during which odourless air was delivered. Participants were instructed to control their eye blinking as much as possible.

EEG setting

EEG was recorded during stimuli presentation from Cz, according to the international 10–20 system using an 8-channel amplifier (Nihon Kohden, Tokyo, Japan) referenced to linked earlobes (A1, A2). Blink artefacts were monitored from an additional recording site (Fp2). Stimulus-linked EEG-segments of 2048 s were digitally recorded at a frequency of 250 Hz (low-pass filter 15 Hz). For recordings, pre-analysis was applied using Letswave 5 software (http://www.nocions.org/letswave5/). To establish baseline, recordings started 200 ms prior to stimulus onset. The epochs were inspected by eye for any kind of artefacts (blinks, muscle contractions etc.). Epochs containing amplitudes of 100 µV or higher were discarded. Clean epochs were baseline corrected, also filtered with a low-pass filter (30 Hz) and averaged in the time

domain. Then, the peak amplitudes (N1, P2, n1p2) and peak latencies (ln1, lp2) were measured using Letswave 5 software.

Behavioural testing

The ability to discriminate between the two enantiomers was assessed using 9 trials of a 3-AFC discrimination test. The participants had to discriminate the target odour [(+)-limonene from two identical non-target odours [(-)-limonene)]. The stimulus duration was set at 500 ms with an inter-stimulus interval (ISI) of 6s and approximately 20s (±4) between presentations of each triplet. Subsequently, participants were also asked to provide ratings of odour intensity using a computerized visual analogue scale (VAS) ranging from 0 to 100 units (0 - "stimulus not perceived" to 100 - "very intense").

Results

Behavioural data

All subjects showed a normal sense of smell with an average TDI score of 37.3 points (SD: 2.3). The average discrimination performance for the target odour was 6.2 (SD: 1.3). Applying a one sample t-test, this result differed significantly from chance level (t [11]) = 8.66, $p < 0.001$). Regarding the intensity ratings, no significant differences emerged between the two enantiomers [∅ intensity (+)-limonene: 53.4 (SD: 13.4), (-)-limonene: 53.5 (SD: 13.4); t [11]) = 0.02, $p = 0.98$) as showed by the paired sample t-test.

Electrophysiological data

We found a significant difference between the two enantiomers regarding AP2 [(+)-limonene 11.29 (SD: 2.46), (-)-limonene 9.4 (SD: 2.9); t [11]) = 2.44, $p = 0.033$) (Figure S1). No differences

emerged for the other components by means of the paired sample t-test (all $p_s > 0.05$) [AN1 (in μV): (+)-limonene -3.37 (SD: 2.88), (-)-limonene -3.52 (SD: 3.1); AN1P2: (+)-limonene 14.67 (SD: 3.7), (-)-limonene 12.92 (SD: 4.01)] and latencies [TN1 (in ms): (+)-limonene 623.17 (SD: 66.38), (-)-limonene 624.17 (SD: 54.92); TP2: (+)-limonene 812.58 (SD: 39.5), (-)-limonene 808.17 (SD: 56.34)].

--- Please insert Figure S1 about here -

REFERENCES

[1] Hummel, T., Sekinger, B., Wolf, S. R., Pauli, E. & Kobal, G. 'Sniffin' sticks': olfactory performance assessed by the combined testing of odor identification, odor discrimination and olfactory threshold. *Chem. Senses* **22,** 39–52 (1997).

[2] Laska, M. & Teubner, P. Olfactory Discrimination Ability of Human Subjects for Ten Pairs of Enantiomers. *Chem. Senses* **24,** 161–170 (1999).

Figure Legend

Figure 1S. Grand average of OERP in response to (+)-limonene (red line) and (−)-limonene (blue line).

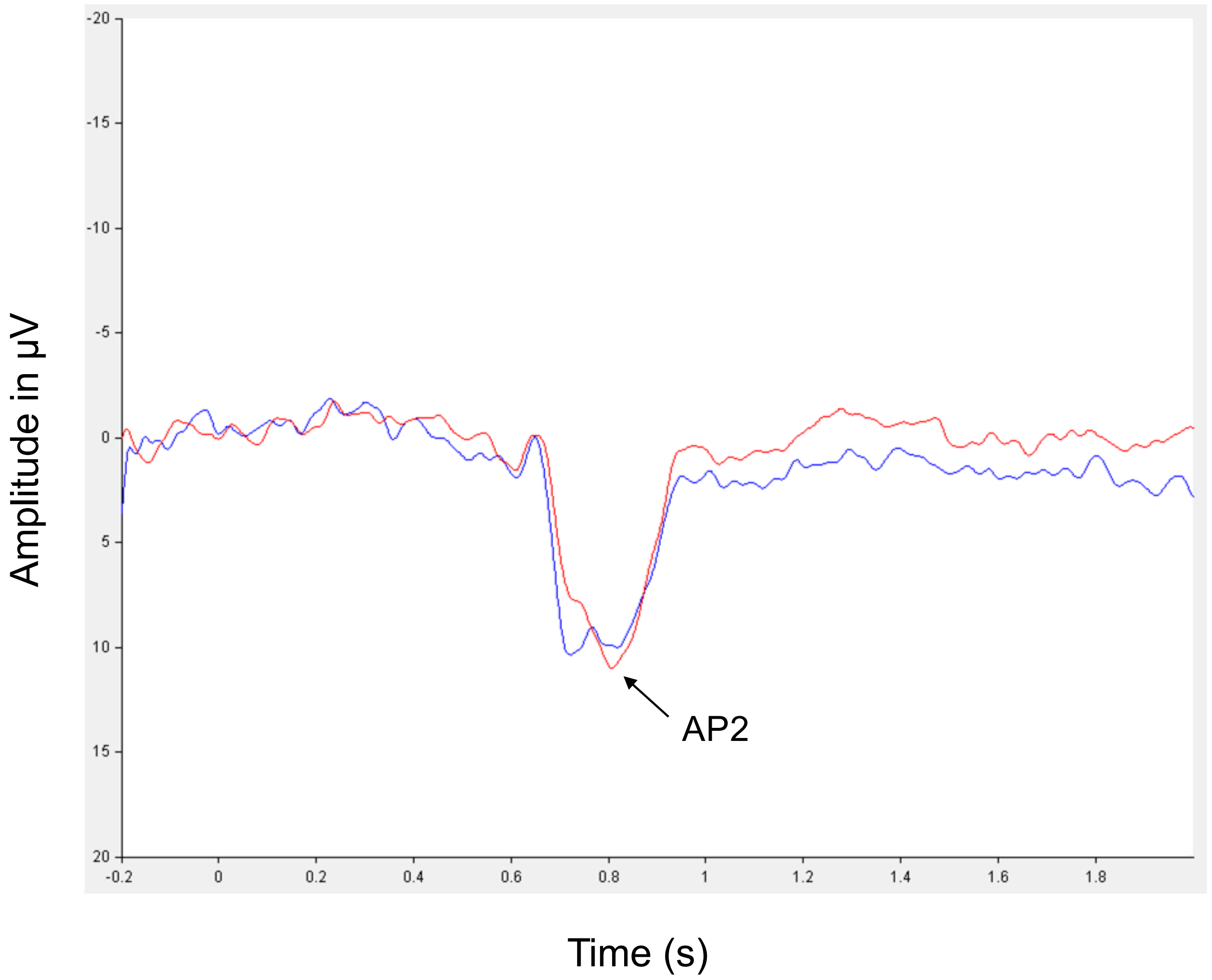

Amplitude in µV
-20
-15
-10
-5
0
5
10
15
20
-0.2
0
0.2
0.4
0.6
0.8
1
1.2
1.4
1.6
1.8
AP2
Time (s)
